# A Chemical Chaperone Restores Connexin 26 Mutant Activity

**DOI:** 10.1021/acsptsci.3c00056

**Published:** 2023-06-01

**Authors:** Dahua Wang, Hongling Wang, Lu Fan, Tobias Ludwig, Andre Wegner, Frank Stahl, Jennifer Harre, Athanasia Warnecke, Carsten Zeilinger

**Affiliations:** † Gottfried-Wilhelm-Leibniz University of Hannover, BMWZ (Zentrum für Biomolekulare Wirkstoffe), Schneiderberg 38, 30167 Hannover, Germany; ‡ Gottfried-Wilhelm-Leibniz University of Hannover, Institut für Technische Chemie/BMWZ (Zentrum für Biomolekulare Wirkstoffe), Callinstr. 5, 30167 Hannover, Germany; § Clinic for Otorhinolaryngology Surgery, Hannover Medical School (MHH), 30625 Hannover, Germany; ∥ Technische Universität Braunschweig, Braunschweig Integrated Centre of Systems Biology (BRICS), Department of Bioinformatics and Biochemistry, Rebenring 56, 38106 Braunschweig, Germany

**Keywords:** Cx26, protein microarray, hearing impairment, viability assay, thermophoresis

## Abstract

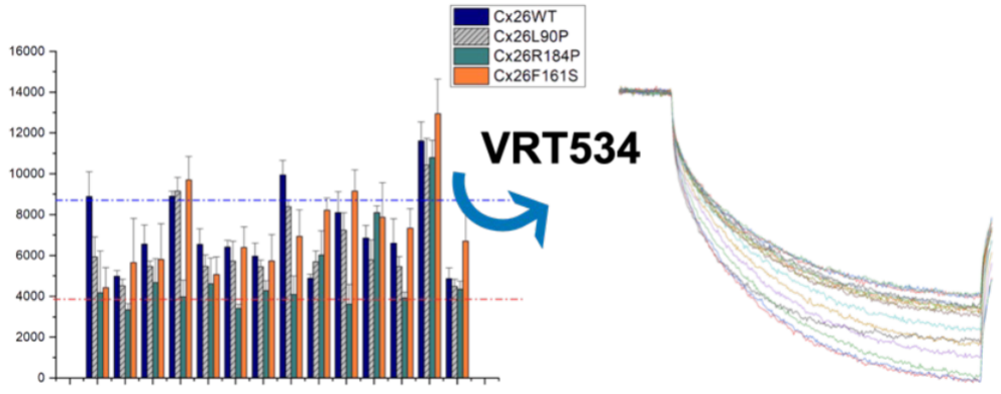

Mutations in connexin 26 (Cx26) cause hearing disorders of a varying degree. Herein, to identify compounds capable of restoring the function of mutated Cx26, a novel miniaturized microarray-based screening system was developed to perform an optical assay of Cx26 functionality. These molecules were identified through a viability assay using HeLa cells expressing wild-type (WT) Cx26, which exhibited sensitivity toward the HSP90 inhibitor radicicol in the submicromolar concentration range. Open Cx26 hemichannels are assumed to mediate the passage of molecules up to 1000 Da in size. Thus, by releasing radicicol, WT Cx26 active hemichannels in HeLa cells contribute to a higher survival rate and lower cell viability when Cx26 is mutated. HeLa cells expressing Cx26 mutations exhibited reduced viability in the presence of radicicol, such as the mutants F161S or R184P. Next, molecules exhibiting chemical chaperoning activity, suspected of restoring channel function, were assessed regarding whether they induced superior sensitivity toward radicicol and increased HeLa cell viability. Through a viability assay and microarray-based flux assay that uses Lucifer yellow in HeLa cells, compounds **3** and **8** were identified to restore mutant functionality. Furthermore, thermophoresis experiments revealed that only **3** (VRT-534) exhibited dose-responsive binding to recombinant WT Cx26 and mutant Cx26K188N with half maximal effective concentration values of 19 and ∼5 μM, respectively. The findings of this study reveal that repurposing compounds already being used to treat other diseases, such as cystic fibrosis, in combination with functional bioassays and binding tests can help identify novel potential candidates that can be used to treat hearing disorders.

Hearing loss is one of the most common neurodegenerative diseases, affecting up to 7% of the global population (>460 million people). According to the World Health Organization, the cost of treating hearing loss is approximately >750 billion dollars annually.^1,2^ Approximately 3.8 million people in Germany live with untreated hearing loss, with a cost of the treatment of ∼40 billion euros annually.^3^ The causes of hearing loss are manifold, including genetic predisposition, ototoxic substances, environmental influences, aging processes, and immunological causes.^4^

Connexin is the only protein family known to form gap junction, thereby affording direct intercellular communication.^5^ In humans, the connexin family comprises 21 different proteins. Among the connexin family proteins, Cx26 (coded by the gene gap junction beta 2 [*Gjb2*]) and Cx30 (coded by the gene gap junction beta 6 [*Gjb6*]) are expressed in the cochlear tissue, responsible for K^+^ recycling in the endolymph of the inner ear. Cx26 mutations constitute the most common form of genetic defects that induce nonsyndromic hearing loss.^6−9^

Gap junction channels comprise two opposing hemichannels of a plasma membrane,^10,11^ with each hemichannel being composed of six subunits and each subunit having four transmembrane domains. Cx26 is an active hemichannel, and the gap junction formed is responsible for cell–cell communication.^12−14^ The Cx26 hemichannel is permeable to small molecules up to 1.2 kDa in size.^12,15^

To date, >100 different Cx26 mutations have been described, with new variants being continually discovered.^16−19^ Different genetic disorders or variants produce varying phenotypes.^18−20^ For example, the truncated variant 35delG induces the complete loss of the connexin function,^21,22^ whereas, in the presence of the M1V, P173R, and R184P mutations, Cx26 protein is not produced.^9,23^ Furthermore, connexins with M34T, L90P, R127H, or F161S do not function or are not correctly transported and incorporated into the cell membrane despite their high expression levels.^23,24^ There are two possible effects of Cx26 mutations: the misfolding and removal of the protein or its incorporation into the plasma membrane in the inactive form.^25−28^ Similar phenomena were described in cystic fibrosis transmembrane conductance regulator (CFTR), and other proteins have been developed to restore those functionalities.^29−31^ The functional hemichannel properties of wild-type (WT) Cx26 have been characterized via voltage clamp experiments, single-channel analyses, molecular dynamic analyses, Raman measurements, and cell-based microarray experiments.^32−35^ Studies have reported that Cx26 mutations affect its hemichannel activity and are thermosensitive.^32,33^ In a previous study, we used HeLa cells as an expression system for WT Cx26 and its mutated forms to investigate the channel function of WT Cx26 and mutants R127H, R184P, and F161S.^33^

Herein, we investigated the effect of antiHsp90 and other compounds on the viability of HeLa cells expressing WT Cx26^36^ or Cx26 mutants to identify compounds that can restore Cx26 function affected by mutations. As a repurposing strategy, known compounds were preselected and assessed to determine if they mediated the restoration of microarray-based thermosensitive dye transport. Finally, the identified candidates were analyzed using thermophoresis to evaluate their interaction with Cx26 and their effect on metabolism and cell viability.

## Results

**Scheme 1:**
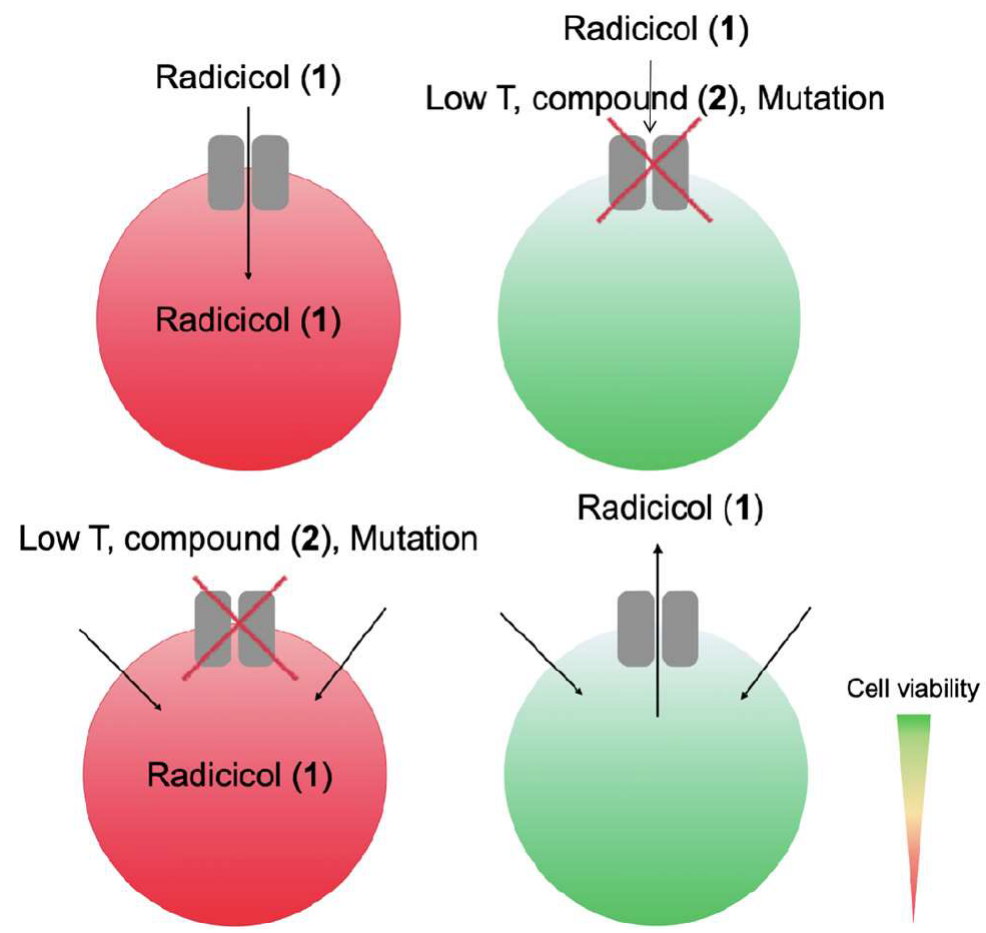
Effect of Radicicol (**1**) on HeLa Cells Expressing WT Cx26 and Cx26 Mutants Upper row, (left) radicicol inhibits Hsp90 and blocks cell survival, thereby reducing cell viability (red), when radicicol is transported via WT Cx26 (gray) only. Upper row, (right) Cx26 is blocked by low temperature, carbenoxolone (a known gap junction inhibitor) (compound **2**), or a mutation, which does not allow radicicol to enter, thereby increasing cell survival and viability (green). Lower row, (left) when radicicol entry into the cell is independent of Cx26, it enters the cell and inhibits cell survival, thereby reducing cell viability; therefore, gap junction inhibitors or mutation have no influence. Cell viability is then observed when radicicol is released through active or restored Cx26 (right, green).

**Scheme 2:**
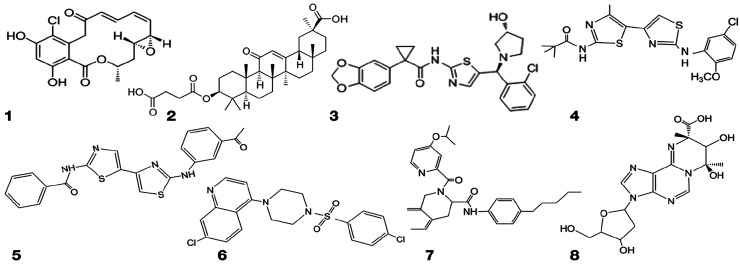
Chemical Structures of Radicicol (**1**), Carbenoxolone (**2**), and Chemical Chaperones (**3**–**8**)

**Figure 1:**
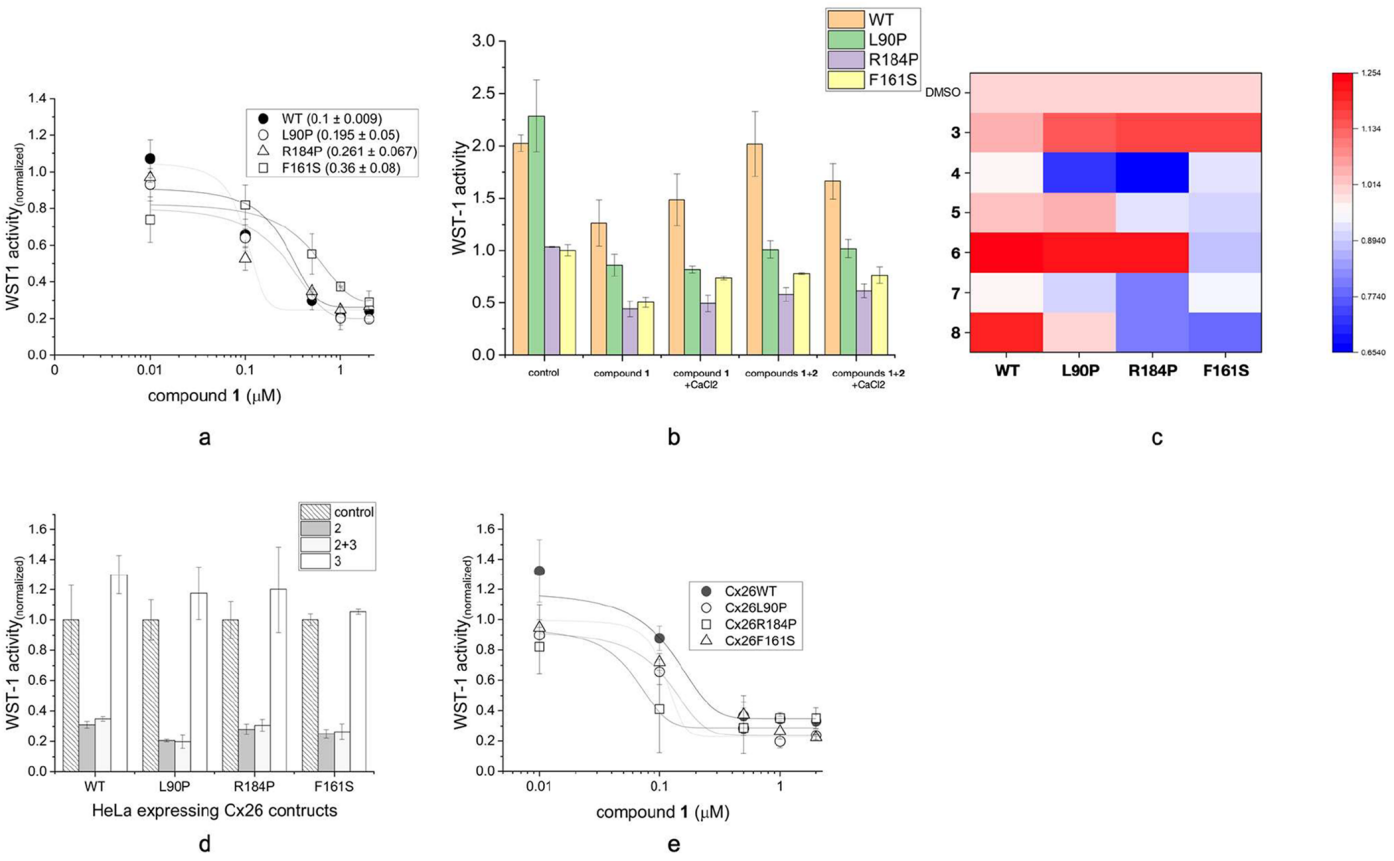
(a) Effect of radicicol (**1**) on the viability of HeLa cells expressing WT Cx26 and Cx26 mutations (L90P, R184P, and F161S). The cells were precultivated for 24 h and then incubated for 24 h with **1** (1 μM). Cell proliferation was assessed via the WST1 assay following an additional 24 h incubation with medium. The data were fitted using a dose–response function to calculate the IC_50_ values for susceptibility to **1**. (b) Effect of radicicol (1 μM) on HeLa cells expressing WT Cx26 or Cx26 mutations (L90P, R184P, and F161S) in the presence of connexin-blocking compounds. The WST1 assay was performed following 30 min of preincubation with or without 0.5 mM carbenoxolone (**2**), 2 mM Ca^2+^, or 2 mM Ca^2+^ and **2**. The data are given as mean and SD of three experiments. (c) Effect of putative chaperoning compounds on HeLa cells expressing WT Cx26 and Cx26 mutations (L90P, R184P, and F161S). The WST1 assay was performed after 1 h of preincubation with **2**–**7** or 1% dimethyl sulfoxide (DMSO) as control. The normalized activity data are visualized as a heatmap with a value of 1 (presented in red) indicating normalized activity in DMSO and 0 (presented in blue) indicating no activity. (d) Functional restoration of Cx26 by **2** in HeLa cells expressing WT Cx26 or Cx26 mutations (L90P, R184P, and F161S). Bar histogram of the WST1 assay performed after 1 h of preincubation with **1**, **2**, **1** + **2**, and 1% DMSO (control). The activity was normalized in DMSO treatment. The data are given as mean and SD of three experiments. (e) Effect of restoring mutant activity and radicicol sensitivity of HeLa cells expressing WT Cx26 and Cx26 mutations (L90P, R184P, and F161S). The cells were precultivated for 24 h and then incubated for 1 h with **3** (50 nM); then, **1** (1 μM) was added, and the sample was incubated for another 24 h. Cell proliferation was assessed via the WST1 assay following an additional 24 h of incubation with medium. The data were fitted using a dose–response function to calculate the IC_50_ values for susceptibility to **1**. The data are given as the mean and standard deviation (s.d.) of three experiments.

Preliminary work involving Cx26-expressing HeLa cells printed on microarrays revealed that the thermosensitive properties of the hemichannel remain preserved on the microarray, which can be useful for evaluating the effect of mutations on the transport of dyes through the channel.^33^ As HeLa cells are cancer cells, cell viability and effect of Hsp90 inhibitors can be optically determined via a dye assay, which can also be used as a proliferation assay.^37^ Hsp90, a cell stress and tumor marker, serves as a target for anticancer drugs owing to its high accumulation in cancer cells.^38^ Hence, we assessed the effect of antiHsp90 compounds, such as radicicol, on cell viability. In our earlier experiments, we revealed that mutated Cx26-expressing HeLa cells exhibit a much lower viability than WT Cx26-expressing cells. Therefore, we concluded that Cx26 channels are potentially responsible for clearing toxic compounds generated by the high turnover of cell metabolites, which contribute to the increased viability of the WT Cx26-expressing cells compared to the Cx26 mutation-expressing cells.^39−43^ Furthermore, Cx26 hemichannels can release ATP, K^+^, Ca^2+^, and dyes, thus contributing to paracrine signal transduction and influencing inflammatory processes. However, whether Cx26 hemichannels are involved in drug release remains unknown. Although it has been suggested that connexins are not required for antiHsp90 compound uptake, the exact uptake route remains unclarified. We hypothesized that Cx26 hemichannels contribute to the import of Hsp90 inhibitor drugs into HeLa cells, thereby reducing cell viability, whereas Hsp90 inhibitors increase cell viability or produce no effect in cells with mutated connexins (Scheme 1). Consequently, factors such as temperature or Cx26 inhibitors (Scheme 2) also affect cell viability in the presence of radicicol (**1**), a well-known antiHsp90 inhibitor (Figure 1a).

After seeding, the cells were incubated for 24 h and then treated with **1** at indicated concentrations for 24 h, followed by an additional incubation of 24 h in fresh media. Afterward, a water-soluble tetrazolium salt (WST1) assay was performed. Figure 1a depicts the effect of **1** on the viability of the HeLa cells expressing WT Cx26 or mutants. Furthermore, WT Cx26-expressing cells exhibited an increased sensitivity to **1**, indicating that Cx26 hemichannel transports **1** across the membrane and Cx26 mutations reduce the hemichannel function. Compound **1** reduced the viability of WT Cx26-expressing HeLa cells in a dose-responsive manner, reducing 50% cell viability at ∼100 nM **1**, whereas Cx26 mutation-expressing cells exhibited relatively weak susceptibility to **1**, sufficient to inhibit proliferation. Overall, the data indicated that Cx26 mutation-expressing cells exhibited reduced viability; however, the half maximal inhibitory concentration (IC_50_) values of **1** against Cx26 mutation-expressing cells were similar to those against WT Cx26-expressing cells. Earlier, immune blotting experiments demonstrated that only the Cx26L90P mutant exhibited a remarkable Cx26 expression, whereas Cx26 mutants R184P and F161S exhibited a weak expression, probably owing to the degradation of mutated Cx26.^43^

To investigate whether blocking Cx26 activity can reduce susceptibility to **1**, a proliferation assay was performed in the presence of carbenoxolone (**2**) and/or Ca^2+^, which are known Cx26 inhibitors. Figure 1b depicts the inhibition of half maximal proliferation by **1** and a reduced sensitivity to radicicol in the presence of **2** and/or Ca^2+^ in the Cx26 mutation-expressing cells. The effect of Ca^2+^ was much stronger than that of **2**, and the cell viability reducing effect was mostly apparent in WT Cx26-expressing cells.

Furthermore, the restoration effects of the preselected compounds (**3**–**8**) on the function of Cx26 mutants were investigated via proliferation assays. Chemical chaperones have been successfully used in treating cystic fibrosis caused by CFTR mutations to transform mutated channels into their functional forms.^29−31^ Cell proliferation in HeLa cells expressing WT Cx26 or Cx26 mutations was examined via proliferation assays. The results revealed that **4**, **5**, **7**, and **8** inhibited cell proliferation in all Cx26-expressing cells and **6** inhibited cell proliferation only in Cx26F161S mutation-expressing cells. Compound **3**, known as the chemical chaperone VRT-534, did not demonstrate any inhibitory effect on the proliferation of cells expressing WT Cx26 or Cx26 mutations (Figure 1c). Conversely, **3** restored the proliferation activity of Cx26 mutation-expressing cells to the same level as that of WT Cx26-expressing cells (Figure 1d). In the presence of 50 nM **3**, the Cx26 mutation-expressing cells exhibited the same degree of sensitivity toward **1** as that of WT Cx26-expressing cells (Figure 1e). Hence, we concluded that **3** has a restorative effect on Cx26 mutants L90P and F161S.

**Figure 2:**
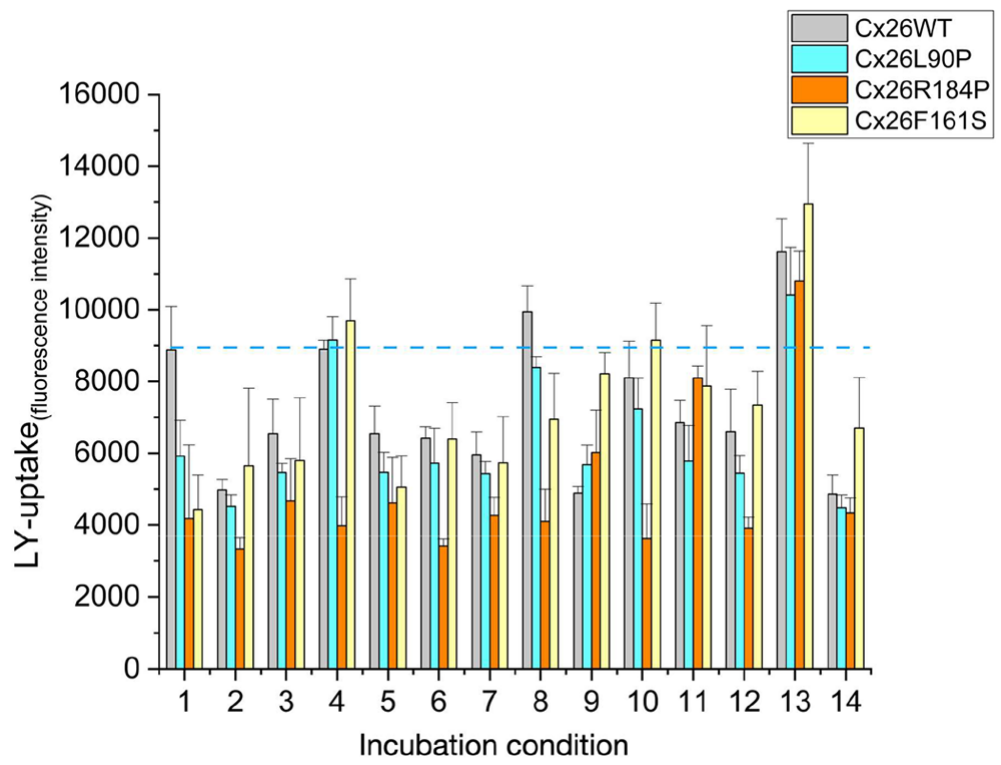
Microarray-based LY uptake assay in microsomes obtained from HeLa cells expressing WT Cx26 or Cx26 mutations. Microsomes were spotted on the NC microarray in columns of eight spots and were preincubated for 1 h at 4 °C with or without **3**–**8** at 2 or 20 μM concentration as indicated and with **2** at 0.5 mM and subsequently incubated with 2% LY. LY uptake as fluorescence intensities are presented as a function of experimental condition (microsome type and compound concentration) and given as the mean of eight spots with the standard deviation (s.d.). Compound **2** was used as an inhibitor control. The data are given as mean and SD of eight spots using the following incubation conditions: 1 = control; 2 = compound **2**; 3 = compound **3** (2 μM); 4 = compound **3** (20 μM); 5 = compound **4** (2 μM); 6 = compound **4** (20 μM); 7 = compound **5** (2 μM); 8 = compound **5** (20 μM); 9 = compound **6** (2 μM); 10 = compound **6** (20 μM); 11 = compound **7** (2 μM); 12 = compound **7** (20 μM); 13 = compound **8** (2 μM); 14 = compound **8** (20 μM).

Next, a Lucifer yellow (LY) flux assay was performed using microsomes isolated from HeLa cells (microsome flux assay) expressing WT Cx26 or Cx26 mutations (L90P, R184P, and F161S) to investigate the direct restorative effect of the compounds on Cx26 activity, as described recently.^33^ Microsomes isolated from HeLa cells expressing WT Cx26 or Cx26 mutations (L90P, R184P, and F161S) were spotted in columns of 10 spots on a nitrocellulose (NC) surface. After transfer to an incubation chamber, single pads of the microarray were preincubated with buffer containing different chemical chaperones at indicated concentrations and subsequently incubated with LY at 37 °C. The effect of preselected chemical chaperones and **2** on the signal intensity of LY inside the microsomes obtained from HeLa cell lysates expressing WT Cx26 or Cx26 mutations is shown in Figure 2. Cx26 mutations and **2** reduced LY uptake (Figure 2).

**Figure 3:**
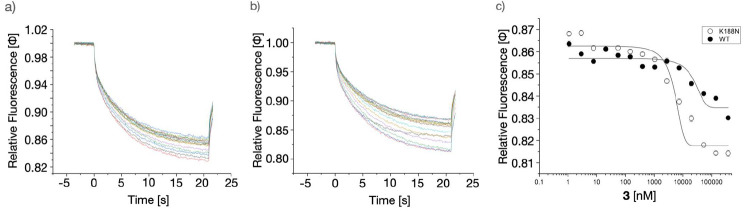
Analysis of affinity of **3** toward Cx26 via MST. Monitoring the interaction between WT Cx26 or Cx26K188N and **3** via MST. MST traces of Cy5-labeled WT Cx26 (a) and Cx26K188N (b) with increasing concentrations of **3** are displayed in the mode of thermophoresis + T_–jump_. Different concentrations from 0 to 50 μM of **3** are shown in different colors of traces. Laser-induced temperature changes for F_cold_ were from −1 to 0 s and those for F_hot_, from 4 to 5 s. (c) The data were dose–response fitted using the function *y* = *A*_1_ + (*A*_2_ – *A*_1_)/(1 + 10^(log *x*_0_–*x*)×*p*^) between the top and bottom asymptotes, with hill slope *p* and log *x*_0_ as the center of the indicated concentration *x*. Dose-responsive fitting of the WT Cx26 and Cx26K188N for **3** induced an affinity of 19.33 ± 7.3 μM for WT Cx26 and 4.75 ± 1 μM for Cx26K188N obtained from two independent experiments and standard deviation (s.d.).

The compounds **3** and **8** restored LY uptake in Cx26 mutants. Compound **3** restored the activity of Cx26L90P and Cx26F161S to that of the WT Cx26, and **8** restored the activity of all mutants (dashed blue line). Herein, we presented a microarray-based technique that can identify compounds and restore channel function as well as demonstrate the direct influence of the protein–compound interaction on the transport activity of Cx26. In addition, a thermophoresis assay was performed to estimate the affinity of **3** toward WT Cx26 and Cx26K188N (as described earlier).^33^ The recombinant connexin channel, purified as described earlier,^33^ was dialyzed against 1× phosphate buffer saline and labeled using a His-tag Cy5 labeling kit. The labeled protein was transferred to a microscale thermophoresis (MST) apparatus to assess the binding affinity of **8** to purified WT Cx26 (Figure S1). The thermophoresis experiments revealed that higher concentrations of **3** exhibit a moderate effect on the affinity toward WT Cx26 and Cx26K188N, with dose–response values of 19.3 ± 7.3 and 4.75 ± 1 μM, respectively, indicating that, alongside a structural function, the mutations induce a higher binding affinity as a precondition for restorative activities (Figure 3a–c).

**Figure 4:**
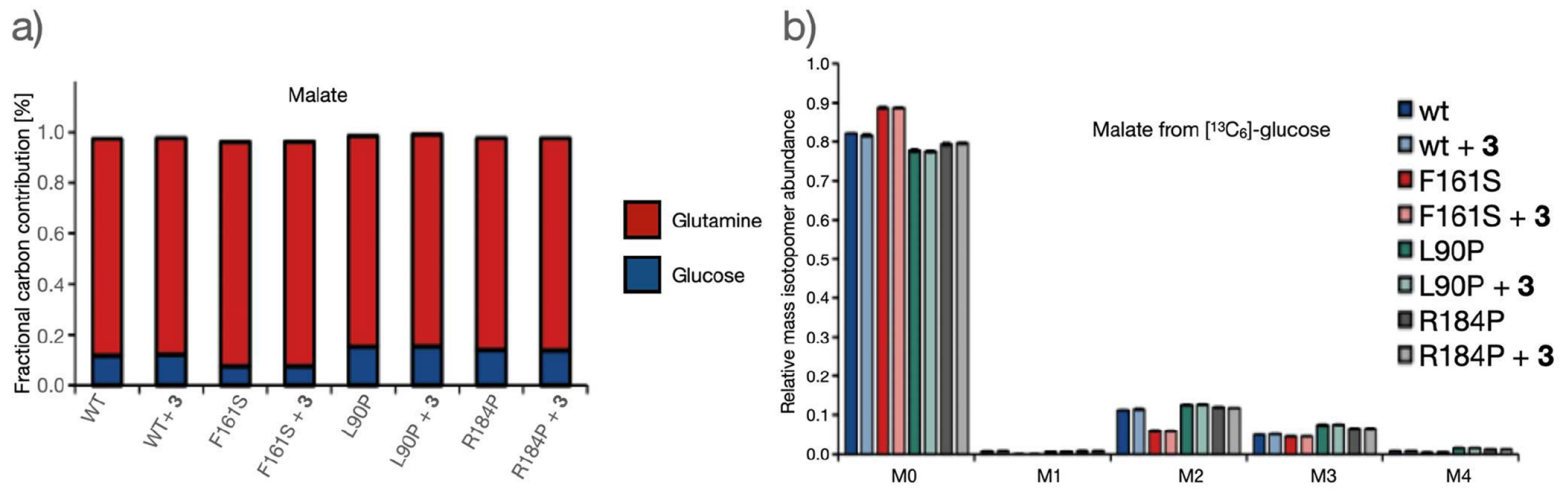
Stable isotope labeling analysis of WT Cx26 and Cx26 mutants. (a) Relative glutamine and glucose carbon contribution to malate production, calculated using the equation  where *n* denotes the number of carbons and *m*_i_ denotes the *i*^th^ mass isotopomer. (b) Mass isotopomer distribution of malate from [^13^C_6_]glucose. The F161S mutant exhibited a significant decrease in M2 malate isotopologs. As M2 malate is produced from [^13^C_2_]acetyl-CoA, glucose catabolism via the pyruvate dehydrogenase complex (PDC) was indicated to be reduced. Mass isotopomer distribution of malate from [^13^C_5_]glutamine is shown in Figure S1. The data are given as the mean and standard deviation (s.d.) of three experiments.

A combination of viability and microarray-based flux assays alongside MST was used to identify the restorative effects of the compounds on channel activity. Furthermore, assays on the central carbon metabolism were performed to evaluate the consequences of channel dysfunction and restoration. Thus, we performed stable isotope labeling experiments using [^13^C_6_]glucose and [^13^C_5_]glutamine and quantified the isotopic enrichment in terms of mass isotopomer distributions. The results revealed decreased glucose carbon contribution to the tricarboxylic acid (TCA) cycle in the Cx26F161S mutant compared with WT Cx26 and other Cx26 mutants (Figure 4a). Subsequently, we investigated whether the decreased glucose contribution was due to reduced anaplerosis via pyruvate carboxylase (PC) or reduced pyruvate dehydrogenase complex (PDC) activity. Using [^13^C_6_]glucose as a tracer, the two pathways were distinguished by analyzing the M2 and M3 isotopologs of TCA cycle intermediates. Briefly, PDC activity generated [^13^C_2_]acetyl-CoA, from which M2 malate was obtained, whereas PC activity yielded [^13^C_3_]oxaloacetate, producing M3 malate. A significant decrease in M2 malate in the Cx26F161S mutant cells was observed, suggesting reduced glucose catabolism via PDC (Figure 4b). Conversely, an increased glutamine carbon contribution to the TCA cycle was observed (Figure 4a). In line with the decreased M2 malate production from [^13^C_6_]glucose, we observed increased M4 malate production from [^13^C_5_]glutamine, suggesting increased glutaminolysis in the Cx26F161S mutant (Figure S1). Although the differences are small, compound **3** was not able to restore or influence the [^13^C_6_]glucose mediated transport of Cx26F161S, which may indicate that glucose processing is not influenced.

## Discussion

This study established a microarray assay technique to identify the compounds that can help overcome the disruption caused by single point mutations in Cx26.^33^ This strategy alongside a viability assay assisted in identifying the preselected compounds that can induce susceptibility toward antiHsp90 compounds in HeLa cells and revealed that correctly expressed and functioning connexin channels may contribute to increased cell viability. Toxic metabolic compounds are possibly cleared from intracellular spaces via connexin hemichannels. Therefore, WT Cx26-expressing cells were found to exhibit higher viability than Cx26 mutation-expressing cells. However, the latter were also susceptible to radicicol, an Hsp90 inhibitor (Figure 1a). Blocking WT Cx26 reduced the viability of HeLa cells, similar to unblocked mutants R184P and F161S (Figure 1b), thereby indicating that connexins contribute to the viability of HeLa cells. As a small molecule, radicicol can be transported through hemichannels, akin to LY that exhibits a similar molecular mass. Thus, this assay was used to screen preselected compounds from an in-house library that can restore the susceptibility of HeLa cells to radicicol when the connexin hemichannel is active. These compounds belong to the category of molecules that have been previously assessed for other targets, such as CFTR. Channel function restoration has been suspected of being mediated via correctors, potentiators, or inhibitors by binding to the protein, hindering misfolding, or quenching false contact sides. This preselection helped evaluate smaller entities of molecule libraries. The restorative ability of **3**–**8** was assessed on the viability of Cx26 mutation-expressing HeLa cells, and **3** was found to restore the viability of such HeLa cells, whereas other compounds, such as **8**, restored the function of only some mutants, such as L90P and R184P. All other compounds were less effective (Figure 1c,d). Compound **3** restored the sensitivity of mutants to radicicol close to or better than that of WT Cx26 (Figure 1e). The microarray-based flux assay confirmed that **3** and **8** restored the flux activity of mutated Cx26.^33^ A more detailed analysis confirmed that the binding affinity for Cx26, identified via thermophoresis, may be responsible for mediating restorative activity. A similar effect was described for **3** (VRT-534), which is used to treat cystic fibrosis by specifically targeting the G551D mutation.^44^ Although no related structure or mutation exists in Cx26, the binding of compounds with moderate affinity using a repurposing strategy can help in further identifying molecules exhibiting high affinity or be used for screenings in molecular docking experiments.

## Experimental Procedures

### Materials

Compound **1**, (1a*R*,2*E*,4*E*,14*R*,15a*R*)-8-chloro-9,11-dihydroxy-14-methyl-1a,14,15,15a-tetrahydro-6*H*-oxireno[e][2]benzoxacyclotetradecine-6,12(7*H*)-dione (Radicicol), and compound **2**, 3β-hydroxy-11-oxoolean-12-en-30-oic acid 3-hemisuccinate were purchased by Merck. Compounds **3**–**8** were kindly provided by the CFTR compound program 2018: compound **3**, 1-(benzo[*d*][1,3]dioxol-5-yl)-*N*-(5-((*S*)-(2-chlorophenyl)((*R*)-3-hydroxypyrrolidin-1-yl)methyl) thiazol-2-yl) cyclopropanecarboxamide (VRT534); compound **4**, *N*-(2-(5-chloro-2-methoxyphenylamino)-4′-methyl-4,5′-bithiazol-2′-yl)pivalamide (C17); compound **5**, *N*-(2-(3-acetylphenylamino)-4′-methyl-4,5′-bithiazol-2′-yl)benzamide (C13); compound **6**, 7-chloro-4-(4-(4-chlorophenylsulfonyl)piperazin-1-yl)quinoline (C9); compound **7**, 2-(4-isopropoxypicolinoyl)-*N*-(4-pentylphenyl)-1,2,3,4-tetrahydroisoquinoline-3-carboxamide (C7); compound **8**, (7*R*,9*S*)-7,8-dihydroxy-3-(4-hydroxy-5-(hydroxymethyl)tetrahydrofuran-2-yl)-7,9-dimethyl-3,7,8,9-tetrahydropyrimido[1,2-i]purine-9-carboxylic acid (B4). All compounds mentioned above are dissolved in DMSO, and the DMSO concentration did not exceed 1% in the test.

### Proliferation Assay

HeLa cells expressing Cx26WT or mutants were transferred to a 96-well microplate (Nunclon Delta Surface, Thermo Scientific, Waltham, USA) with a cell concentration of 2.5 × 10^5^ cells/mL and incubated for 24 h at 37 °C and 5% CO_2_. The compounds (radicicol) or substances were added with the final concentration as indicated, and the cells were incubated for 1 h in the presence of 1.8 mM EDTA. The inhibitors were removed; fresh medium was added, and the cells were incubated for another 24 h. The medium was then removed, and the cell proliferation reagent WST-1 (10%, Roche) was added and incubated for half an hour. The measurements were performed in a microplate reader (Synergy H1 Hybyid Reader, BioTek). The wavelength for measuring the absorbance of the formazan product is 450 nm. The reference wavelength is 630 nm.

### Preparation of Microsomes for Microarray Flux Assay (MFA) and Lucifer Yellow Uptake Assay (LYUA)

Cultivated HeLa cells (4 × 10^6^ cells/mL) with Cx26WT or mutated Cx26 were concentrated and resuspended in a PBS buffer containing protease inhibitor, and microsomes were isolated as described recently.^32^ The suspension was transferred three times through a 10 cm-long injection needle. Afterward, the homogenate was centrifuged at 10 000*g* for 10 min at 4 °C and the supernatant was sedimented at 50 000*g* for 30 min at 4 °C. Next, the sediment was resuspended in 100 μL of TBS. The LYUA was performed as described earlier.^31^ The vesicles were centrifugated for 30 min at 5000*g* or filtrated through a 0.2 μm filter and spotted as described before onto the nitrocellulose membrane of a microarray utilizing a contact-free piezoelectric nanoplotter (NanoplotterNP2.1, GeSim) using around 20 droplets per spot and spotting parameters as follows: 100 Hz, 50 μs, and 80 V; one droplet was equal to the volume of 0.8 nL. The pads were incubated with EM buffer (120 mM NaCl, 7 mM KCl, 0.8 mM MgCl_2_, 5 mM glucose, and 25 mM Hepes at pH 7.3) containing 2% Lucifer yellow for 15 min at 30 °C, and free dye was removed by three exchanges with EM buffer and incubated 10 min. The supernatant was removed, and the fluorescence signals were analyzed by using a Laser Scanner (SensoSpot Fluorescence and Colorimetry Microarray Analyzer, Miltenyi Biotec Company, Germany) using blue illumination (410–480 nm wavelength) for 5 s; fluorescent signals were calculated with the provider’s preinstalled array analysis software (Sensospot). Data were analyzed and performed using OriginPro 11 (OriginLab Cooperation).

### Microscale Thermophoresis (MST)

Cx26WT and Cx26K188N in buffer containing 1× PBS were incubated in darkness for 30 min. Cx26WT and Cx26K188N were labeled according to a Cy5 His tag labeling protocol (Nano Tempr), and a stock solution of 50 μL (1 mg/mL) was adjusted to a final concentration of 50 nM. To estimate signal intensity, a prerun was performed at a protein concentration of 50 nM in an MST glass capillary, and the proper LED power on Monolith NT.115 was checked to yield low florescence between 200 and 1500 counts. For the stock solution, a 12 step dilution series of compound **3** in MST buffer containing 1× PBS from 0.01 to 2000 nM was prepared. The ratio between each of two adjacent dilution steps was 1:1 at a final volume of 10 μL. Ten μL of labeled Cx26WT solution was filled into the same 12 tubes and mixed very well. The mixed sample was incubated in the dark at room temperature for 20 min. After an adequate incubation time, all the samples were transferred into Monolith NT capillaries. The capillaries were inserted into the slots on the sample tray, and the measurements were started. The measurements were performed at a LED power of 30% using a MST power of 20%, 40%, and 60% and a final protein concentration of 50 nM at different concentrations of compound **3**. Afterward, the capillaries were scanned and the MST measurement started with three repetitions. Data analysis and calculation of the dose responsive affinity was done by NT analysis software.

### Metabolomic Analysis

HeLa cells were seeded in 6-well plates with 100 000 cells per well. After 24 h of incubation, the medium was exchanged with medium containing ^13^C6 labeled glucose (25 mM) or ^13^C5 glutamine (4 mM) and the 12C analogue compound. Instead of FBS, 10% dialyzed FBS was used. For the treatment, 50 nM VRT534 was added to the medium. The cells were incubated for 24 h before the metabolite extraction was performed. After washing the cells with 0.9% NaCl, 400 μL of methanol (−20 °C) and 400 μL of dH_2_O (4 °C) containing 1 μg/mL glutaric-*d*_6_ acid as internal standard were added to the cells. Subsequently, the cells were detached from the surface by scraping. The suspension was transferred to a tube containing 400 μL of chloroform (−20 °C) and agitated for 20 min at 1400 rpm and 4 °C. To separate the polar and nonpolar phase, the samples were centrifuged for 5 min at 17 000*g* and 4 °C. 250 μL of the polar phase was transferred to a GC glass vial and dried overnight at 4 °C using a refrigerated vacuum concentrator. After capping, the samples were applied to GC-MS analysis.

Prior to the measurement, the samples were derivatized with 15 μL of a 2% methylhydroxylamine solution in pyridine and agitated for 90 min at 55 °C. In a second step, 15 μL of MTBSTFA was added and the sample was agitated for additional 60 min. After that, the samples were transferred to an Agilent 7890A GC equipped with a 30 m DB-35MS + 5 m Duraguard capillary column connected to an Agilent 5975C inert XL MSD. All measurements were performed using selected ion mode.^42^ The subsequent data analysis with chromatogram processing and calculation of mass isotopomer distributions was performed with the Metabolite Detector software.^45^

## Supporting Information Available

Analysis of the M4 malate measurement from [^13^C_5_]glutamine (PDF)

## Abbreviations

Cx26: connexin 26
Hsp90: heat shock protein 90
LY: Lucifer yellow
DMSO: dimethyl sulfoxide
