# Supplementary material for: A Chemical Chaperone Restores Connexin 26 Mutant Activity
Source: ACS Pharmacol Transl Sci. 2023 Jun 1;6(7):997–1005. doi: 10.1021/acsptsci.3c00056 (PMC10353060; doi:10.1021/acsptsci.3c00056)
Supplement: Supplementary file 1 — pt3c00056_si_001.pdf [file pt3c00056_si_001.pdf]

A chemical chaperone restores connexin 26 (Cx26) mutant activity

Dahua Wang[1,3], Hongling Wang[1,3], Lu Fan[1,3], Tobias Ludwig[4], Andre Wegner[4], Frank Stahl[2], Jennifer Harre[3], Athanasia Warnecke[3], Carsten Zeilinger[1*]

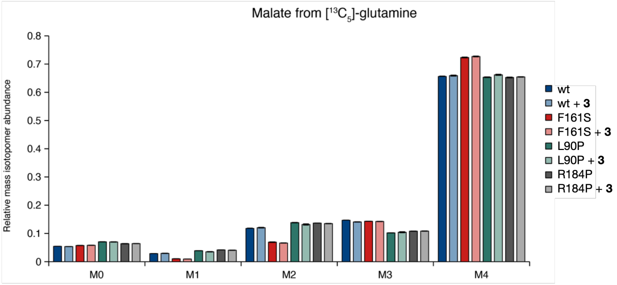

Figure S1: Mass isotopomer distribution of malate from [$^{13}C_5$]-glutamine. The F161S mutant shows increased M4 isotopologues, pointing towards an increased glutaminolysis.
